# In Memoriam: Professor Paolo Muiesan (1961–2022)

**DOI:** 10.3389/ti.2022.10595

**Published:** 2022-05-31

**Authors:** Constantino Fondevila, Luciano Potena, Umberto Cillo, Gabriel Oniscu, the ELITA Board, R. Adam

**Affiliations:** ^1^ Hospital Universitario La Paz & La Paz Research Institute (IdiPAZ), Madrid, Spain; ^2^ Centro de Investigaciones Biomédicas de la Red de Enfermedades Hepáticas y Digestivas, Madrid, Spain; ^3^ Heart Failure and Transplant Unit, IRCCS Bologna Academic Hospital, Bologna, Italy; ^4^ General Surgery, University of Padova, Padua, Italy; ^5^ Edinburgh Transplant Centre, Edinburgh, United Kingdom

**Keywords:** DCD, transplant, liver, split, ELITA

The transplant and surgical communities remain shocked and saddened by the recent passing of Professor Paolo Muiesan (1961–2022), renowned hepatopancreatobiliary and transplant surgeon and beloved father and friend.

Professor Muiesan was internationally recognized for his incredible technical skill as well as his diligent focus on research, advancing the evidence and pushing the limits of what could be done in liver transplant and HPB surgery. For many of us, he was also “Paolo”—dashing, affable, and above all kind. He talked to everyone and did not mind being called upon by anyone for help, to give a brilliant talk, to offer his thoughts on a difficult case, or just to discuss the ups and downs of life.

In a field of many dedicated professionals, it is hard to imagine anyone who worked harder than Paolo Muiesan. He performed highly complex surgical procedures skillfully and efficiently, and his hands were integral in saving the lives of thousands of adult and pediatric patients. He was generous and trained many individuals who are now surgeon leaders in their own right. He talked at countless meetings, organized conferences, and led the field by chairing numerous professional groups and societies. He played a particularly important role in the European Liver and Intestine Transplant Association (ELITA), joining the Council in 2007, serving as Secretary from 2008–2012, and ultimately rising to the role of Chair from 2012–2015. He also provided the voice and representation of the European Society for Organ Transplantation (ESOT) in the European Union of Medical Specialists (UEMS). Most recently, he was elected to the Council of the International Liver Transplantation Society (ILTS).

While Paolo Muiesan distinguished himself in many areas related to liver transplant and HPB surgery, perhaps his greatest and most consistent contribution was as a “founding father” of donation after circulatory death (DCD) liver transplantation. Where few dared to venture, Paolo persevered; without his careful, consistent effort, the panorama of DCD liver transplant and organ transplantation in general would be nowhere close to where it is today.

Paolo Muiesan performed his medical school and surgical training in Milan and Brescia. He then left Italy for many years, first establishing himself at Kings College Hospital in London and subsequently moving to Birmingham at the Queen Elizabeth Hospital and Birmingham Children’s Hospital, where he also held a personal chair at the University of Birmingham. He had returned Italy in 2018, working in Florence and most recently at Policlinico in Milan since Fall 2021. Anyone who talked to him in the past year knows how excited he was to finally be home, to embark on new challenges and share his incredible knowledge and skill base in his “terra natia.” The irony of his passing at the pinnacle of his professional career and in the moment of his homecoming carries a particularly biting sting.

We are all saddened by his loss, but the impact of Paolo’s passing is greatest felt by those that were closest to him in life. Professional accomplishments aside, Paolo was first and foremost a father to two beautiful boys, now both young men. Paolo was proud of his sons, Andrea and Matteo, and talked about them frequently. The thoughts and support of our entire community remain with them at this difficult time.

Paolo was an unstoppable “forza della natura” until he left us; his life force now lives on in all that he leaves behind. Paolo’s legacy lies in the hundreds of journal articles and books he published as well as the numerous colleagues with whom we worked and helped train. Above all, Paolo’s legacy is the two lives he created and the countless others he worked to save.

Ciao, Paolo, you are gone far too soon, but you will never be forgotten.


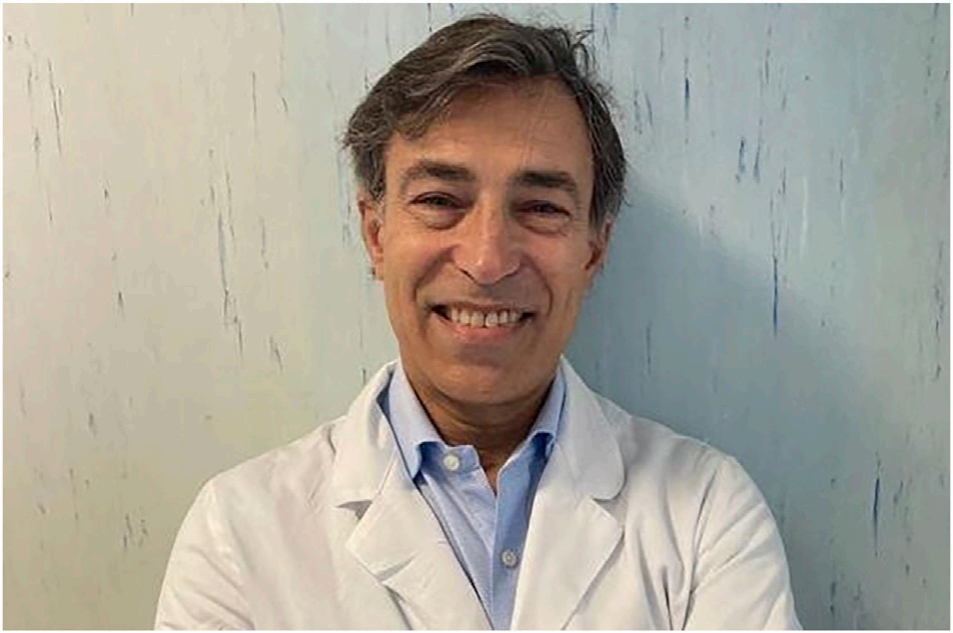


## ELITA Board Members

R. Adam, U. Baumann, L. Belli, G. Germani, H. Hartog, S. Nadalin, W. Polak, P. Taimr, C. Toso, and K. Zieniewicz

